# Common (Mis)Beliefs about Memory: A Replication and Comparison of Telephone and Mechanical Turk Survey Methods

**DOI:** 10.1371/journal.pone.0051876

**Published:** 2012-12-18

**Authors:** Daniel J. Simons, Christopher F. Chabris

**Affiliations:** 1 Department of Psychology and Beckman Institute, University of Illinois, Champaign, Illinois, United States of America; 2 Department of Psychology, Union College, Schenectady, New York, United States of America; Goldsmiths, University of London, United Kingdom

## Abstract

Incorrect beliefs about memory have wide-ranging implications. We recently reported the results of a survey showing that a substantial proportion of the United States public held beliefs about memory that conflicted with those of memory experts. For that survey, respondents answered recorded questions using their telephone keypad. Although such robotic polling produces reliable results that accurately predicts the results of elections, it suffers from four major drawbacks: (1) telephone polling is costly, (2) typically, less than 10 percent of calls result in a completed survey, (3) calls do not reach households without a landline, and (4) calls oversample the elderly and undersample the young. Here we replicated our telephone survey using Amazon Mechanical Turk (MTurk) to explore the similarities and differences in the sampled demographics as well as the pattern of results. Overall, neither survey closely approximated the demographics of the United States population, but they differed in how they deviated from the 2010 census figures. After weighting the results of each survey to conform to census demographics, though, the two approaches produced remarkably similar results: In both surveys, people averaged over 50% agreement with statements that scientific consensus shows to be false. The results of this study replicate our finding of substantial discrepancies between popular beliefs and those of experts and shows that surveys conducted on MTurk can produce a representative sample of the United States population that generates results in line with more expensive survey techniques.

## Introduction

A substantial proportion of the United States public holds beliefs about memory that conflict with well-established expert consensus. In a nationally representative telephone survey with a nominal sample of 1500 people, on average 60.4% of respondents agreed with six statements that were almost universally rejected by a sample of memory experts [1]. This nationally representative sample produced results comparable to earlier telephone surveys of jurors and police as well as classroom and in-person surveys of students and the public [2], [3], [4], [5], [6], [7], showing that mistaken intuitions about memory are pervasive [8], [9]. For decisions that rely on intuitions about what people should remember and how well they should remember it, these mistaken beliefs can have devastating consequences. For example, according to the Innocence Project, many of those exonerated from death row following DNA testing were convicted on the basis of flawed eyewitness testimony, and jurors evaluate the credibility of that evidence based on their often mistaken understanding of the workings of memory [6].

Of the many techniques used to produce a representative sample of a population, random-digit-dialing telephone surveys are among the best established and most widely used. With appropriate weighting, such surveys can produce a valid estimate of the percentage of a target population holding a common belief. For that reason, nationally representative samples are a crucial test of the generalizability of results from the local samples commonly used in studies of mistaken intuitions. Telephone surveys have many drawbacks, though: (1) nationally representative telephone samples can cost many thousands of dollars, (2) commonly, less than 10 percent of calls result in a completed survey, (3) polls typically do not call cell phones due to the greater expense involved, and relatedly (4) calls oversample the elderly and undersample the young. Often large samples are needed to compensate for deviations from the target demographic distribution, making it necessary to weight some respondents more heavily than others.

Amazon Mechanical Turk (MTurk) provides a new way to conduct large-sample surveys that bypass some of the limitations of telephone surveys. MTurk is a system in which “workers” complete online jobs (Human Intelligence Tasks, or HITs, in MTurk jargon) posted by “requesters” (e.g., companies or researchers). In this case, the requester is the researcher and the workers are the subjects or respondents. After a worker completes a HIT, the requester instructs Amazon.com to credit the worker’s account. Amazon.com handles all transactions so that workers remain effectively anonymous to the requesters. MTurk now has more than 500,000 registered workers throughout the world.

To our knowledge, though, no studies have yet compared the representativeness and results from a large sample of MTurk workers to those of a nationally representative telephone survey using random-digit dialing. In this study, we replicated our national telephone survey on beliefs about psychology [1] with a large MTurk sample, using the same items. This sample allowed us to compare the demographics of these two polling methods. Moreover, by weighting the results of both surveys to match the 2010 census demographics, we could compare the rates of agreement with our survey items. To the extent that the MTurk results match those of our telephone survey, the results would provide evidence that MTurk can be used to conduct nationally representative surveys, and would also constitute a replication of our initial results.

Since its inception in 2005, psychology researchers have been using MTurk to norm their stimuli and to conduct survey studies. Several studies have surveyed MTurk workers to assess their demographic profile and found the samples to be more representative than typical university subject pools or online samples [10], [11]. The demographic data provided by participants also tend to be reliable [12]. Those workers responding to HITS asking for their demographic information tend to be somewhat better educated than the broader United States population, with somewhat more female respondents than male ones. Although there have been concerns about the validity of online data collection in psychological research, especially due to the self-selected nature of samples of web participants [13], several studies have compared the outcomes of laboratory-based experiments to data collected from MTurk, and in most cases, the results have been comparable [10], [14], [15]. In this study, we explore the sampled demographics from MTurk and compare them to those from our telephone survey that used the same questionnaire [1].

By weighting both samples to census demographic figures, we found that these two approaches to nationally representative surveys produce roughly comparable patterns of results. Together, the results of our original survey, coupled with this replication using a different sample and survey technique, show the extent to which common beliefs deviate from long-established expert consensus. In both surveys, respondents average more than 50% disagreement with a scientific consensus that, in some cases, has been established for decades. The prevalence of these misconceptions in our samples suggests that many people likely rely on faulty intuitions about the workings of the mind when making practical decisions that affect their own and others’ lives [1], [6]; students may rely on faulty intuitions about the accuracy and permanence of memory when deciding how to study, and jurors may falsely convict innocent people of crimes because they rely on similarly mistaken beliefs about the accuracy of witness recollections.

## Methods

### Ethics Statement

All data were collected anonymously, and the study conformed to the guidelines and principles of the Declaration of Helsinki. The protocol was approved by the University of Illinois Institutional Review Board and was given a waiver of the requirement for signed consent because it is an anonymous online survey.

Between July 4 and August 3, 2011, we repeatedly posted a HIT on MTurk entitled “Beliefs about Psychology” which was described as “Answer a short survey about psychology.” By reposting the HIT 11 times we hoped to attract respondents who might not have seen the initial posting. The text instructions informed potential respondents that we were “conducting a short opinion survey of your beliefs about psychology.” After reading a consent screen informing them that they would be paid $0.25 for completing the survey and that they could cease participation at any time, they followed a link to SurveyMonkey to complete the survey. At the end of the survey, the final screen provided a code that participants could then enter on MTurk to receive payment. The survey was restricted to adult participants from the United States, and it included safeguards so that only one participant could respond from a given IP address (to make it harder for the same person to participate repeatedly).

This survey used the same items, wording, and ordering as our earlier telephone survey [1], and it consisted of 16 substantive statements about psychology and the mind, with each worded to be inconsistent with the scientific consensus. Here we compare the results for the six items related to memory that were reported in our earlier article about our telephone survey. Other unreported survey items addressed beliefs about other topics in psychology, such as the myth that people use only 10% of their brain or that notion that vaccines cause autism. The memory items were interspersed among the other items.

Each statement appeared on its own page in bold font, and participants clicked on the appropriate radio button to indicate whether they “Strongly Agree,” “Mostly Agree,” “Mostly Disagree,” “Strongly Disagree,” or “Don’t Know.” After responding, they clicked the “Next” button to proceed to the next statement, and they could not return to a previous page once they had advanced. Following the substantive items, respondents provided the same demographic information as in the original survey, including: age, sex, race, state of residence (to determine their region), household income, education, and experience with psychology books and psychology classes. The entire survey took approximately 5 minutes to complete, on average. Of the 1020 people who began the survey, 982 completed it.

## Results and Discussion

Our earlier phone survey found high rates of agreement with statements that counter established scientific consensus, revealing widespread misconceptions about the workings of memory [1]. We found the same pattern with our MTurk sample, with roughly comparable levels of agreement for all our items. Before comparing the results of the two surveys, we address the central question of this study: What is the relative efficiency and accuracy of the sampling procedure achieved via telephone surveys and MTurk? To that end, we first compare the raw demographics of the samples in each survey.

### Comparison of Raw Sample Demographics

The SurveyUSA data were collected in 2009, so they were based on the 2000 United States Census distribution. SurveyUSA constrained their random-digit dialing to obtain a sample that was representative of the regional population distribution at that time, separated into four regions (Northeast, South, Midwest, and West). The MTurk data were collected in 2011, but no effort was made to sample in proportion to the U.S. Census figures for region. Consequently, we might expect the SurveyUSA sample to be more representative of the regional distribution in the United States. Surprisingly, though, the MTurk sample came closer to approximating the distribution according to the 2010 U.S. Census demographics (Table 1). Both surveys slightly oversampled the Northeast and Midwest and undersampled the South and West, but the deviations were smaller overall for the MTurk sample. This finding suggests that self-selection for study participation on MTurk approximates the regional distribution of the United States population.

**Table 1:** Unweighted percentage of respondents in each demographic category from MTurk survey and SurveyUSA along with the 2010 US Census targets.

*Category*	*Value*	MTurk	SurveyUSA	Census 2010
Age	18–24	30.52	2.77	13.08
	25–34	30.11	5.17	17.51
	35–44	18.51	8.38	17.52
	45–54	12.41	17.25	19.19
	55–64	7.02	22.09	15.56
	65–74	0.92	20.40	9.26
	75+	0.51	23.94	7.88
Sex	Male	41.30	37.38	48.52
	Female	58.70	62.62	51.46
Race/ethnicity	White	77.72	85.03	66.97
	Black	7.53	7.45	11.65
	Hispanic	5.60	3.16	14.22
	Asian	6.71	1.41	1.93
	Other	2.44	8.50	5.23
Region	Northeast	19.63	18.72	17.65
	South	36.42	37.70	38.78
	Central	22.38	28.18	21.45
	West	21.57	15.40	22.11
Education	Graduate School	20.40	17.01	9.61
	College Graduate	16.46	24.54	18.14
	Some College	36.81	46.13	28.48
	No College	26.33	12.32	43.76
Annual Income	< $40,000	41.77	51.07	–
	$40–$80,000	35.68	32.35	–
	> $80,000	22.55	16.58	–

Note: Some respondents in the Simons & Chabris (2011) SurveyUSA sample did not provide their education or income, so the percentages are out of those who did provide responses.

Both SurveyUSA and MTurk had more women than expected, although the MTurk survey again was closer to the 2010 Census distribution. Both surveys also oversampled White and undersampled Black and Hispanic respondents. The biggest difference between the surveys in their sampled demographics were for age. More than 66.4% of the SurveyUSA sample was over the age of 55, compared to less than 8.5% for the MTurk sample. And more than 60.6% of the MTurk survey was under the age of 35, compared to less than 8.0% for SurveyUSA. In other words, the telephone survey greatly oversampled older participants and undersampled younger participants, and the MTurk survey did the opposite. Therefore, in both cases, obtaining an age-representative sample would require substantial weighting.

Education and Income were not weighted in the original survey, but they do differ somewhat between the surveys as well. For both surveys, approximately one-third of the sample had household incomes in the middle category ($40,000–80,000 per year). But MTurk had proportionately more high-income respondents and fewer low-income participants than the SurveyUSA sample. This finding suggests that the low hourly wages typical of MTurk studies are not exclusively targeting and exploiting low-income individuals, at least for surveys limited to the United States population. Although the MTurk sample had a higher proportion of respondents with a graduate degree, it also had more with no college at all.

### Comparison of Telephone and MTurk Surveys Following Weighting to the 2010 Census

The goal of most surveys is to estimate the proportion of people with particular preferences, attributes, or opinions in the general population. To form a representative sample of the population, pollsters typically weight each of their respondents so that responses from over-sampled groups count less and responses from under-sampled groups count more. These distortions in the distribution of respondents could result from chance sampling error or from other biases. For example, people from some demographics may be more likely to have a landline telephone, to be willing to respond to surveys in general, or to be home to receive the call. To make sure that the sample is large enough to capture all of the critical demographic groups, even those constituting a small proportion of the population, pollsters typically sample a larger number of respondents than the nominal sample size of the survey. In the telephone survey we conducted [1], the total sample consisted of 1838 respondents, and after weighting to account for over- and under-sampling, the final, nominal sample size was 1500.

To compare the proportions of agreement with our survey items across samples, we weighted each sample to match the United States 2010 Census demographics (census data were drawn from www.census.gov/compendia/statab/2012/tables/12s0011.pdf and from Factfinder2.census.gov table QT-P1 Age Groups and Sex: 2010, database 2010 SF1). Given that the MTurk sample was smaller than the telephone survey sample, for comparability we weighted both samples to give a nominal sample size of 750 respondents. In order to have enough respondents in each subgroup, we dichotomized the factors of Age (<44 versus 44+) and Race (white vs. non-white). Among United States adults (18+), the median age is 43.94, so we weighted so that half the nominal sample was <44 and half was 44+. For race, 70.4% of United States adults were white, and 29.6% were another race (including Hispanic). The census found that the adult population consisted of 48.6% men and 51.4% women. The weighted proportions of our nominal 750-person sample, along with the individual response weights, are shown in Table 2. Note that the biggest difference in the weightings across surveys was for age: To compensate for undersampling, the MTurk sample overweighted responses from older participants whereas the SurveyUSA sample overweighted responses from younger participants.

**Table 2:** Proportion of a nominal sample from each respondent category according to the 2010 Census data, along with weights applied to individual respondents in the MTurk and SurveyUSA samples.

*Category*	*nominal sample (%)*	*MTurk weights*	*SurveyUSA weights*
Woman - Young - NonWhite	7.6	0.49140438	1.212827831
Woman - Young - White	18.1	0.435434718	1.191716069
Woman - Old - NonWhite	7.6	4.071636288	0.508954536
Woman - Old White	18.1	1.006338014	0.154733066
Men - Young - NonWhite	7.2	0.664633048	1.993899145
Men - Young - White	17.1	0.505142453	1.379636378
Men - Old - NonWhite	7.2	6.729409613	0.604890752
Men - Old - White	17.1	2.036606082	0.268422977

Note: SurveyUSA weights are based on data from Simons & Chabris (2011), re-normed to 2010 Census data.

Table 3 reports the percentages of participants in each sample agreeing with each memory statement. In both surveys, respondents agreed with an average of approximately 3 of the 6 statements. In other words, they agreed with 50% of the items that experts almost uniformly believed were incorrect [1]. The rates of agreement were roughly comparable across surveys for all 6 items, with none differing by more than 15% of respondents. Those in the MTurk sample endorsed the Amnesia and Inattentional Blindness beliefs more frequently, but they endorsed the Confident Eyewitness, Permanent Memory, and Video Memory beliefs somewhat less often. Overall, then, the MTurk survey produced a pattern of results that was comparable to that from a nationally representative telephone survey.

**Table 3:** Percentage of weighted respondents agreeing with each memory statement, along with the average rate of agreement across items.

*Item*	*MTurk*	*SurveyUSA*
Amnesia: People suffering from amnesia typically cannot recall their own name or identity.	81.4	69.6
Confident testimony: In my opinion, the testimony of one confident eyewitness should be enough evidence to convict a defendant of a crime.	22.1	32.9
Video memory: Human memory works like a video camera, accurately recording the eventswe see and hear so that we can review and inspect them later.	46.9	52.7
Unexpected events: People generally notice when something unexpected enters their fieldof view, even when they’re paying attention to something else.	77.4	65.0
Permanent memory: Once you have experienced an event and formed a memory of it,that memory does not change.	28.0	39.9
Hypnosis: Hypnosis is useful in helping witnesses accurately recall details of crimes.	46.4	44.6
Average agreement rate (out of 6 items)	50.33(3.02)	50.83(3.05)

Note: SurveyUSA data are from Simons & Chabris (2011).

## Discussion

The goals of this study were twofold: (1) to explore whether we could replicate the pattern of mistaken beliefs about memory found in our earlier telephone survey using a different sampling method, and (2) to compare the demographic characteristics of a sample of self-selected Mechanical Turk participants to those of a nationally representative telephone survey to determine whether MTurk could be used to obtain a nationally representative sample of the United States population.

After weighting the samples from both surveys to conform to the same dichotomized version of the 2010 Census figures, the pattern of results for a self-selected MTurk sample closely matched those of our earlier random-digit-dialed telephone survey. Both the overall rates of agreement as well as the relative levels of agreement across items were consistent across samples. Together, these surveys show that a substantial portion of the general population holds mistaken beliefs about how memory works.

These mistaken beliefs have important practical ramifications. First, they show that mistaken beliefs persist in the face of regular efforts to communicate the limits and foibles of memory [6]. The persistence of such mistaken beliefs might be a consequence of our incomplete experiences: We rarely are forced to confront evidence that definitively proves our vivid memories wrong [16]. And, even when mistaken conclusions are corrected, the misconceptions often persist [17]. Second, these misconceptions like these play a role in personal, political, and legal decisions. For example, people are overly trusting in the memory of confident witnesses, perhaps because many people think memory is immutable or more precise than it actually is. Many false convictions are predicated on the mistaken recollections of an eyewitness, and juries convict based on their belief in the veracity of such memories. Only by better understanding the nature and prevalence of such mistaken beliefs can educators and policy makers work to eradicate them.

Although the weighted samples from the two surveys produced comparable results, the unweighted samples from both surveys deviated substantially from the distributions expected based on the census data. Most strikingly, they diverged from the expected age demographics in opposite directions, with the telephone survey massively oversampling the elderly and the MTurk survey massively oversampling the young. After weighting each sample to conform to the United States census demographics, both surveys provided roughly comparable results: In both surveys, people endorsed statements about memory that memory experts almost uniformly dismiss as inaccurate. And, the overall levels of agreement across all 6 items were nearly identical. These findings suggest that studies on MTurk can provide a nationally representative sample using the same weighting procedures used for telephone polling.

Although random digit dialing is considered the gold standard for conducting nationally representative telephone surveys, it suffers from many drawbacks. Telephone surveys are more expensive, they undersample young participants, they have relatively low response rates, and they cannot reach households without landlines (without becoming substantially more expensive). As more households forego landlines in favor of cell phones, traditional surveys risk even greater biases in their sampling. For example, younger participants who have landlines might differ in important respects from those who do not.

No survey method is ideal, and MTurk faces drawbacks as well. MTurk undersamples the elderly about as much as telephone methods undersample the young, and it relies even more on self-selection for study participation. In that sense, it does not provide a random sample of the population. Moreover, the population of MTurk workers is small compared to the pool of potential respondents to a telephone survey, and many MTurk workers participate in many other studies. Consequently, they too might differ systematically from other people with the same demographic characteristics who do not “work” on Mechanical Turk.

Given that both methods have strengths and weaknesses, there is no a priori reason to believe one approach is better or more accurate than the other. Both can produce a nationally representative sample when weighted appropriately to match census demographics. Our approach to doing this has merits for smaller studies as well. Without a sample in the tens of thousands, it would be impossible to obtain enough respondents to fill every demographic sub-category; some categories are sufficiently small in the United States population that the expected number of respondents from that category is effectively 0 without a huge sample (e.g., Asian women living in the south who are between 50 and 60 years of age). Few national surveys are truly representative of population demographics, with proportional numbers of respondents in each sub-category. They must first select demographic factors to consider, and then weight their samples to compensate for over- or under-sampling.

We dichotomized age and race based on the census demographics, and weighted our sample accordingly. The result is a nationally representative sample based on those classifications. That is, our weighting on a dichotomized race variable allows us to generalize to the United States population based on that dichotomy: If we wished to compare survey responses by race, we could compare white and non-white respondents, and our results should generalize to the same dichotomy in the general population. The risk, of course, is that the makeup of one of our sub-categories might not correspond to that of the population (we might have disproportionately more Black respondents than would be expected for the Non-White category in the general population). But any study generalizing from a restricted sample to a larger population faces the same problem [18]. Weighting allows more valid generalization than would result from analyzing the unweighted sample. Moreover, by using MTurk and weighting for national demographics, studies can avoid some of the criticisms involved in generalizing from college undergraduate samples to the population at large [15].

Given that both telephone and MTurk methods have different strengths and weaknesses, but produce roughly comparable results (at least for our memory belief items), they also could be used to complement each other. For example, a survey could draw respondents from both methods and combine them to provide a more representative sample of the age distribution of the United States.
